# Enzyme-free genetic copying of DNA and RNA sequences

**DOI:** 10.3762/bjoc.14.47

**Published:** 2018-03-12

**Authors:** Marilyne Sosson, Clemens Richert

**Affiliations:** 1 Institute of Organic Chemistry, University of Stuttgart, 70569 Stuttgart, Germany

**Keywords:** base pairing, DNA, enzyme-free primer extension, nucleotides, oligonucleotides, replication, RNA

## Abstract

The copying of short DNA or RNA sequences in the absence of enzymes is a fascinating reaction that has been studied in the context of prebiotic chemistry. It involves the incorporation of nucleotides at the terminus of a primer and is directed by base pairing. The reaction occurs in aqueous medium and leads to phosphodiester formation after attack of a nucleophilic group of the primer. Two aspects of this reaction will be discussed in this review. One is the activation of the phosphate that drives what is otherwise an endergonic reaction. The other is the improved mechanistic understanding of enzyme-free primer extension that has led to a quantitative kinetic model predicting the yield of the reaction over the time course of an assay. For a successful modeling of the reaction, the strength of the template effect, the inhibitory effect of spent monomers, and the rate constants of the chemical steps have to be determined experimentally. While challenges remain for the high fidelity copying of long stretches of DNA or RNA, the available data suggest that enzyme-free primer extension is a more powerful reaction than previously thought.

## Introduction

**Figure 1:**
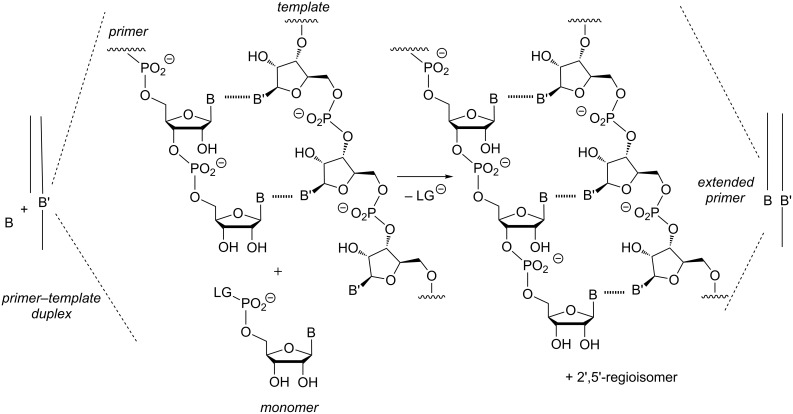
Enzyme-free template-directed extension of an RNA primer by one nucleotide. B = nucleobase, LG = leaving group.

Replication of genetic information is critical for all living systems. In the cell, this process is catalyzed by enzymatic machineries that have polymerases at their core [1]. Polymerases catalyze not only the replication of DNA, but are also involved in repair and transcription of genes [2]. Considering that enzymes catalyze processes that lead to protein synthesis, it is reasonable to ask what started replication when life emerged on planet Earth. A solution to the chicken/egg dilemma of replication might be found in RNA, as oligo- and polyribonucleotides can encode genetic information and can catalyze biochemical reactions as ribozymes. More than 30 years ago, it was observed that RNA strands catalyze splicing or ligation of longer oligonucleotides [3–4]. Ancient ribozymes might have acted as polymerases [5], inducing either the oligomerization of activated ribonucleotides or the replication of the first RNA genomes. But ribozymes are usually too long to be likely to emerge from random sequences in one step. Simple forms may have taken advantage of the high ionic strength of the eutectic phase [6], but their evolution must have been preceded by something simples. In a very simple version of RNA-based replication, genetic copying may have occurred in the absence of both protein enzymes and ribozymes, relying on solely on base pairing for molecular recognition and chemical reactivity to drive the formation of phosphodiester bonds in aqueous media. This is what is usually referred to as "enzyme-free copying" (Figure 1).

**Figure 2:**
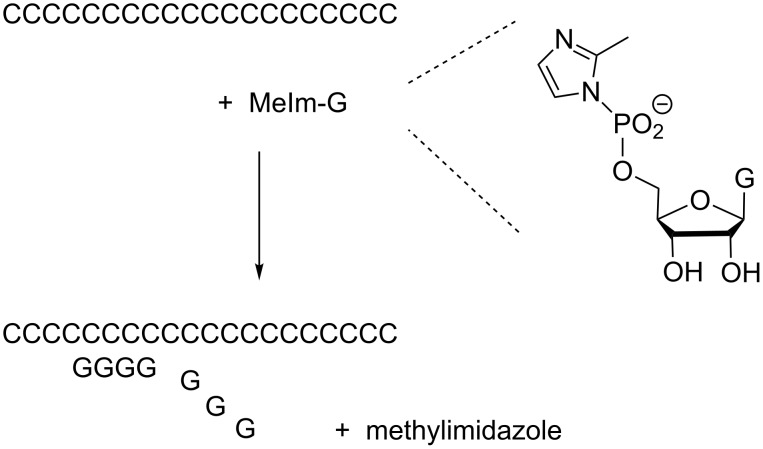
Oligomerization of the 2-methylimidazolide of guanosine-5'-monophosphate on a poly(C) template.

Studies on enzyme-free copying of genetic polymers date back more than 50 years [7]. Classical studies were often focused on ligation reactions, including templated ligations of self-complementary sequences [8–9]. Special systems, such as ligation with triplex-forming sequences [10] have produced some impressive results, and the field of ligation-based replication has been reviewed [11]. Ligation reactions will not be discussed further here, as they are limited in their scope, in terms of sequences, whereas monomer-based copying may be used for any given sequence, at least in principle. Rather, we will focus on copying with mononucleotides, for which early examples can also be found in the literature of the 1960s [12]. The early monomer-based work on copying RNA focused on oligomerization of nucleotides on homosequences as templates [13–14]. The best results were observed for poly(C) as template, the 2-methylimidazolide of guanosine as activated monomer (Figure 2), and assay buffers containing high concentrations of Mg^2+^ ions [15]. When advances in automated solid-phase synthesis made oligonucleotides of any given sequence readily available [16], copying reactions involving the extension of a primer bound to a specific sequence of hairpins mimicking this arrangement became the most common way of performing the reaction [17–20].

**Figure 3:**
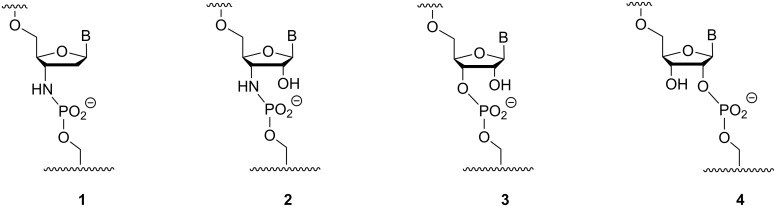
Structures of backbone linkages produced in enzyme-free primer extension reactions: the phosphoramidate of a 3'-amino-2',3'-dideoxynucleoside (**1**), the phosphoramidate of a 3'-amino-3'-deoxynucleoside (**2**), the 3',5'-phosphodiester of a natural ribonucleoside (**3**), and the isomeric 2',5'-phosphodiester of a ribonucleoside (**4**).

In this brief account we will focus on primer extension reactions on DNA and RNA templates. The copying of DNA sequences is usually performed with primers terminating in a 3'-amino-2',3'-dideoxynucleoside. The amino group is much more nucleophilic than the hydroxy group of natural DNA, so that rapid reactions result. Figure 3 shows the structure of the phosphoramidate formed when 3'-aminoterminal DNA primers are extended, together with the phosphoramidate linkage resulting from reactions with 3'-aminoribonucleotides [21], and the two regioisomeric phosphodiesters that result from the extension of RNA primers that terminate in natural ribonucleosides. We note that the phosphoramidate linkages are isoelectronic and largely isosteric to natural phosphodiesters.

In our brief account, we will highlight some of the issues plaguing enzyme-free primer extension. One such issue is incomplete conversion. Many chemical primer extension assays stall long before completion of the reaction, resulting in a mixture of extended and unextended primer. We will then discuss progress in our understanding of the chemical primer extension reaction that was made since our earlier account on the topic [22]. Other reviews that cover enzyme-free copying exist, and the reader is directed to these papers for a more in-depth treatment of issues only touched upon in our account [13,23–25].

## Review

### Template effect and sequence dependence

One factor that significantly affects whether an enzyme-free primer extension reaction occurs in high yield or not is the strength of the template effect. Unlike the reactions that are catalyzed by polymerases, purely chemical primer extension reactions are not facilitated by the active sites of enzymes. Instead, the base pairing between individual bases of an incoming nucleotide and the templating base must suffice to attract the monomer to the extension site. The stability of different base pairs varies, and so does the templating effect of different stretches of the template sequence. Using random homopolymer templates, Joyce and Orgel concluded that the structure and hybridization status of templates was important for high-yielding copying reactions [26]. In a later series of papers with specific, synthetic sequences, Wu and Orgel reported that primer extension proceeds poorly if too many weakly pairing A or U residues are present in the templating sequence [17–19].

**Figure 4:**
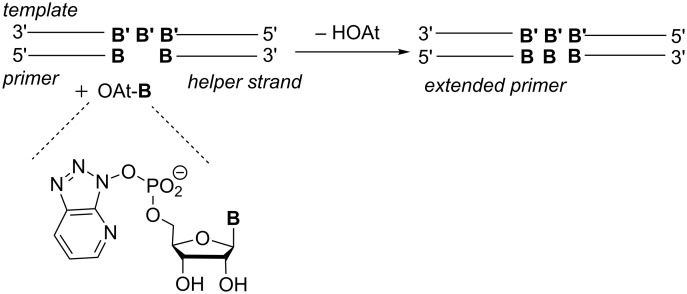
System used for studying the template effect with all 64 possible triplets at the extension site (B/B' = nucleobase).

These experiments had been performed with a riboterminal primer/self-priming hairpin that is low in reactivity. Using more nucleophilic 3'-aminoterminal DNA primers and oxyazabenzotriazolides of deoxynucleotides (OAt-dNMPs) as a more reactive combination than that of the traditional methylimidazolides and RNA primers, we screened all 64 possible base triplets at the elongation site [27] (Figure 4). Both, the base at the center position of the triplet that acts as templating base and either of the flanking bases were varied systematically, and downstream-binding oligonucleotides were tested for their effect. Under these conditions, 90% of the primer was extended successfully in each of the 64 different sequence contexts.

**Figure 5:**
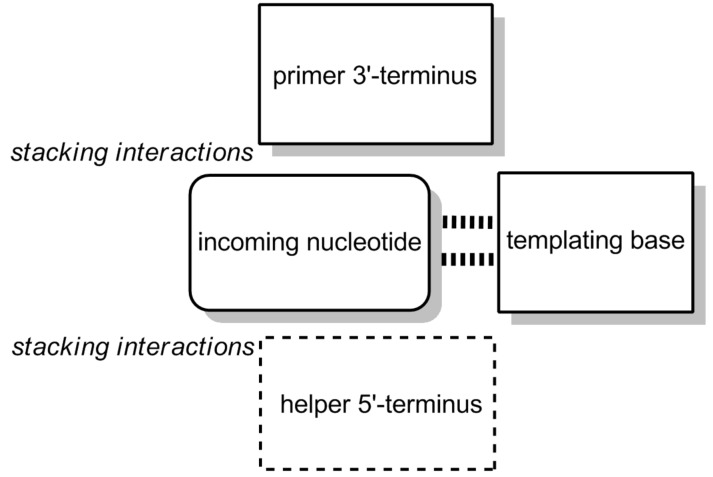
Interactions attracting the incoming nucleotide to the extension site. Besides base pairing via hydrogen bonding to the templating base, stacking interactions with neighboring bases of primer and a possible downstream-binding helper oligonucleotide, as well as help of solvophobic effects, influence the strength of the template effect.

This suggested that the template effect is strong enough to support successful copying, at least when sufficient reactivity exists. When we determined the rates for each of the different templating triplets, we found that the rate constant for extension on the poorest templating sequence (CAG) and on the best templating sequence (TCT) differed by less than two orders of magnitude, with rate constants *k'*_CAG_ = 100 h^−1^ M^−1^ and *k'*_TCT_ = 8 310 h^−1^ M^−1^ [27]. This was encouraging. As expected, the incorporation of G was most favorable, as this base strongly pairs via three hydrogen bonds and has a large surface area for stacking. Numerically, the *t*_1/2_ values for the incorporation of G ranged from 1 min to 15 min, whereas those for T were between 13 min and approx. 2 h under the experimental conditions chosen. Further, a primer terminating in an A residue was found to be favorable. This, the most lipophilic of the bases, probably offers the stickiest stacking surface for incoming bases. When a downstream-binding oligonucleotide is present, stacking with the base of its 5'-terminal nucleoside further adds to the attractive forces experienced by incoming monomers. This is shown schematically in Figure 5. Because downstream-binding strands favorably affect the rate and selectivity of primer extension, we have dubbed them "helper oligonucleotides" [28–29]. Kinetics measured without downstream-binding element were two- to seven-fold slower, depending on the sequence context [27].

Overall, the data mentioned above suggest that there is indeed a strong dependence of the templating base and sequence context on the rate of enzyme-free primer extension assays. Whether the available template effect suffices to induce successful extension in aqueous buffer depends on the reactivity of the nucleophilic group at the primer's 3'-terminus and that of the activated phosphate of the monomer.

### Quantitative model

**Figure 6:**
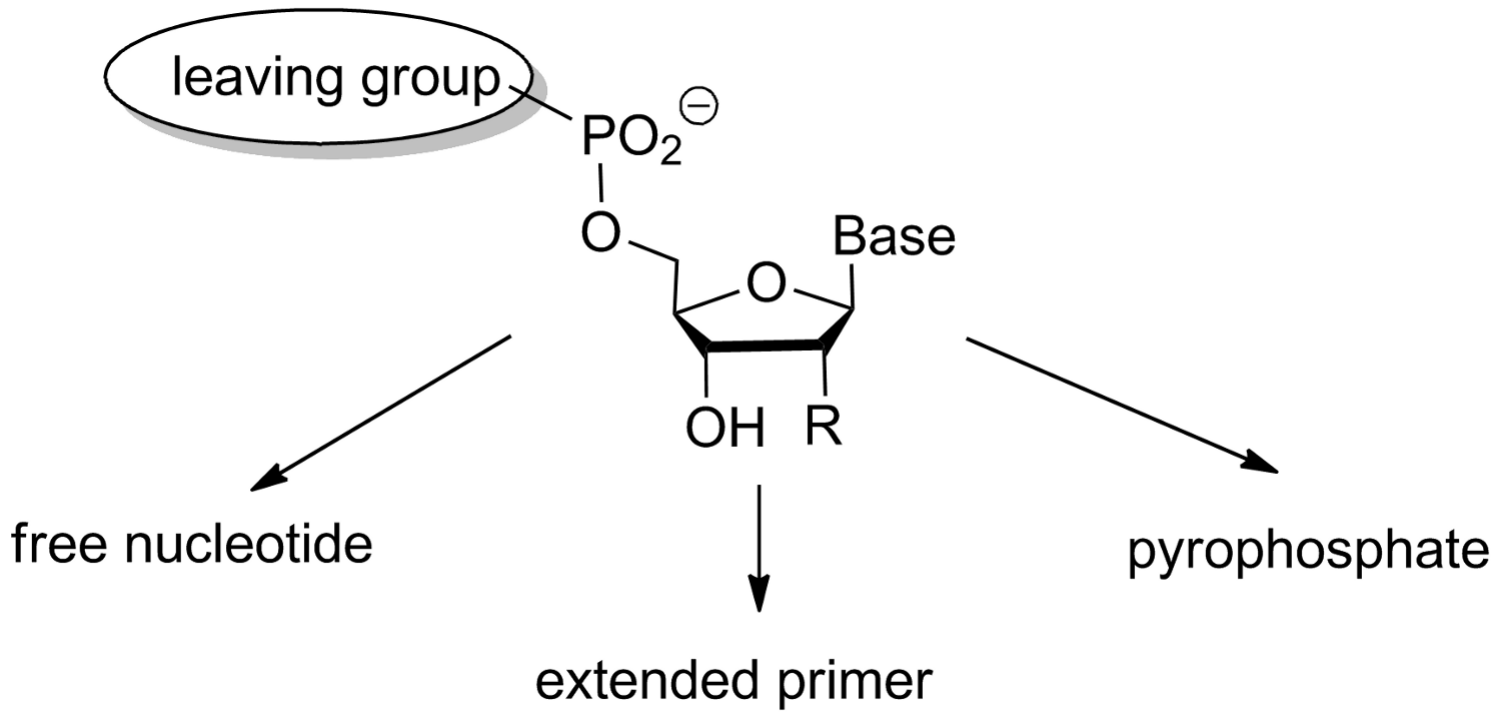
Three possible fates of activated nucleotides in aqueous buffer that result from hydrolysis, primer extension, and reaction with a free nucleotide, respectively. Other possible pathways, such as cyclization to the 3',5'-cyclic diester or oligomerization are not shown graphically; R is OH for ribonucleotides and is H for deoxynucleotides.

To gain a better understanding of the factors responsible for successful or unsuccessful primer extension assays, we embarked on a project aimed at gaining a quantitative understanding of enzyme-free copying. What are the rate- and yield-limiting steps of the reaction? What concentration of the monomer is needed to achieve near-quantitative conversion? Are there other factors that need to be considered to be able to predict the yield of primer extension reactions? These were just some of the questions that motivated this work. We wished to know what the fate of the many nucleotides was that were employed in the assay (usually in large excess over the primer). Figure 6 shows three of the more obvious reaction pathways that came to mind.

Our experimental work used nucleotides pre-activated as oxyazabenzotriazolides (OAt esters, compare Figure 4) [28–29] or as 2-methylimidazolides (MeIm amides, compare Figure 2) [13,22,30]. In aqueous media, hydrolysis of activated nucleotides is all but unavoidable, and hydrolytic deactivation becomes more likely when significant concentrations of magnesium ions are present [31]. High initial concentrations of monomers are usually used to compensate for this problem (0.1 M solutions are not uncommon), but still there is incomplete conversion for extensions that involve incorporation of A or U [19].

Further, it was clear that monomers have to bind to the primer-template duplex prior to experience the template effect and to be incorporated sequence specifically. So, a quantitative understanding of the binding equilibrium was called for. Bimolecular binding equilibria are usually described mathematically via the binding constant or dissociation constant. The latter is more intuitive, as it gives the concentration at which half of the binding partners are in the bound state and the other half is in the free state in an equimolar mixture of the two.

Next, it had become clear from our study on RNA-based copying that the hydrolysis of activated monomers not only reduces the amount of available starting material, but actively lowers reactivity because the hydrolyzed monomer can inhibit primer extension. The hydrolyzed, free nucleotide can still bind to the extension site on the template, and in doing so, prevent the activated form from entering the site, acting as a competitive inhibitor [32]. So, both the rate of hydrolysis and the strength of the inhibitory effect were important factors to be considered in a quantitative model.

**Figure 7:**
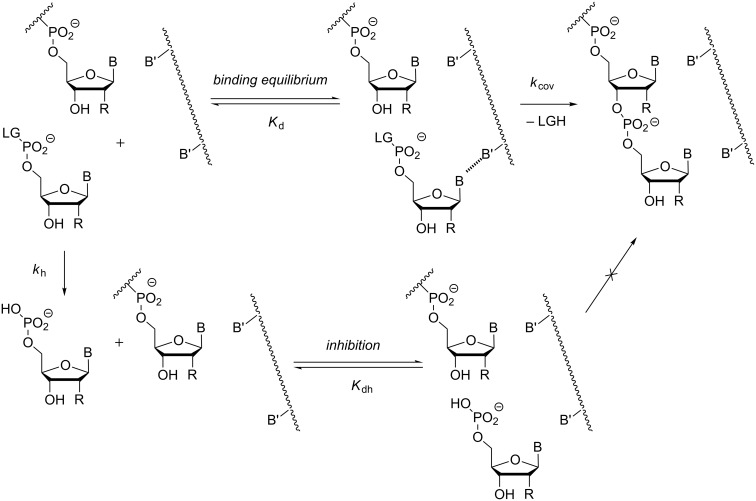
Steps and equilibria considered in our quantitative model of chemical primer extension [34]. The model considers the binding of the activated monomer with its leaving group (LG) to the primer–template complex in the form of the dissociation constant (*K*_d_). It takes into account the rate of hydrolysis with the corresponding rate constant (*k*_h_), the binding equilibrium for the hydrolyzed monomer that acts as inhibitor (*K*_dh_), and it assumes a single rate-limiting chemical step (*k*_cov_); B, B' = nucleobase = OH for RNA.

Figure 7 shows our model for an RNA-based primer extension system. For primer extension to occur, binding between activated nucleotide and primer–template duplex takes place. So, the dissociation constant (*K*_d_) has to be determined experimentally [33]. Once bound, the terminal hydroxy group of the primer has to attack the activated 5'-phosphate of the primer, most likely producing a pentavalent intermediate. Unless the leaving group finds itself in the proper apical position of the intermediate, this is followed by pseudorotation and then the release of the leaving group. Either of these steps can be rate-limiting, and we have encountered two-step kinetics with a lag-phase in some reactions involving aminoterminal primers [27]. More often, though, and in all cases involving ribonucleosides at the 3'-terminus of the primer, kinetics characteristic of a single rate-limiting step are found, so that the modeling requires no more than a single rate constant for the covalent step (*k*_cov_). Determining the rate constant experimentally requires knowledge of *K*_d_, so that a defined concentration of the kinetically relevant species can be entered in the rate equation for what is now a pseudo-first order reaction [33–34]. To properly model the inhibition, both the rate of hydrolysis (*k*_h_) and the dissociation constant of the inhibitor–primer/template complex have to be known. The latter (*K*_dh_) is often similar to the *K*_d_ value for the complex with the activated monomer, so that an approximation assuming this, produces results that are not far off from what modeling with all four constants (*K*_d_, *K*_dh_, *k*_h_ and *k*_cov_) gives [34].

### Hydrolysis of activated nucleotides

In order to gain any insights from the model presented in the preceding paragraph, binding constants and rate constants had to be determined. Among the rate constants was that for the hydrolysis of activated monomers. Hydrolysis was expected to be fast for highly reactive monomers, and the reactivity toward water was expected to be similar to the reactivity toward the terminal diol of an RNA primer, so hydrolysis was considered a very relevant parameter. We focused on the two classes of activated monomers mentioned above: 2-methylimidazolides and oxyazabenzotriazolides. Synthetic methods for producing such monomers from free nucleotides were briefly reviewed in our earlier account [22]. The methylimidazolides were chosen because a large body of literature exists on their reactions including studies by Orgel [35], Kanavarioti [31,36], Szostak [37–38], and Göbel [39]. The oxyazabenzotriazolides are our preferred monomers because they gave us the fastest primer extensions, both on RNA and on DNA templates [28–29,34,40].

Hydrolysis follows pseudo-first order kinetics and is readily measured by ^31^P NMR spectroscopy [28,32–34]. Oxyazabenzotriazolides were indeed found to hydrolyze faster than methylimidazolides, and half-live times of hydrolysis at room temperature for the different nucleotides, in extension buffer containing 80 mM MgCl_2_, were typically found to be in the range of 5–8 h at a pH of 8.9, both for ribonucleotides and for deoxynucleotides. Only OAt-dTMP was slower to hydrolyze, with a *t*_1/2_ of 16 h [28]. Molecular modeling suggested that this may be due to the steric effect of the methyl group at the 5-position of the pyrimidine ring, shielded the leaving group-bearing phosphate from incoming water from some angles of attack. For OAt esters of ribonucleotides, we also measured the rates of hydrolysis at 0 °C and −20 °C, and the detailed data can be found in Supplementary Table S1 of reference [32]. At the lowest of the temperatures assayed, the half-live times increased to values between 51 h for OAt-UMP and 86 h for OAt-CMP.

Methylimidazolides were slower to hydrolyze. The half-lives of hydrolysis for deoxynucleotides were ranging between 19 h and 29 h whereas *t*_1/2_ varied from 53 h to 63 h for ribonucleotides [34]. Our results were thus comparable to the ones obtained by Ruzicka and Frey who studied the hydrolysis of 5'-phosphorimidazolates of uridine at different pH values [41] and found a half-life toward hydrolysis of about 60 h in the absence of Mg^2+^ and at neutral pH, i.e., conditions favoring longevity for this type of activated monomer, which requires protonation of the imidazole ring to be turned it into a good leaving group.

### Binding equilibria

As mentioned above, primer extension involves the binding of the incoming nucleotide to the primer–template complex, being directed by base pairing and stacking interactions. Therefore, it was important to determine the binding constants for activated and unactivated nucleotides experimentally. Theoretical predictions for triphosphates had suggested very tight binding [42], but the strong base dependence of the yield and selectivity of primer extension reactions suggested to us that not all nucleotides occupied the extension site to the same extent.

Initially, we wished to better understand how strong the inhibitory effect of spent monomers was, and we set up experiments to determine the *K*_dh_ value for complexes between free nucleotides and primer–template duplexes. This required methodology adjusted to measuring weak binding, i.e., much weaker than the strand-to-strand hybridization of oligonucleotides leading to duplexes, which is usually monitored by UV-melting analysis [43]. We chose NMR spectroscopy, partly because it is performed at much higher concentrations (millimolar, rather than micromolar analytes), and partly because it provides site-specific information without labeling. Labeling of an analyte as small as a mononucleotide with something other than isotopes was considered problematic, as it would strongly change the structural characteristics, and simple techniques, such as gel shift, do not work for complexes with a fast off-rate because the complex dissociates during the time it takes to perform the electrophoresis.

**Figure 8:**
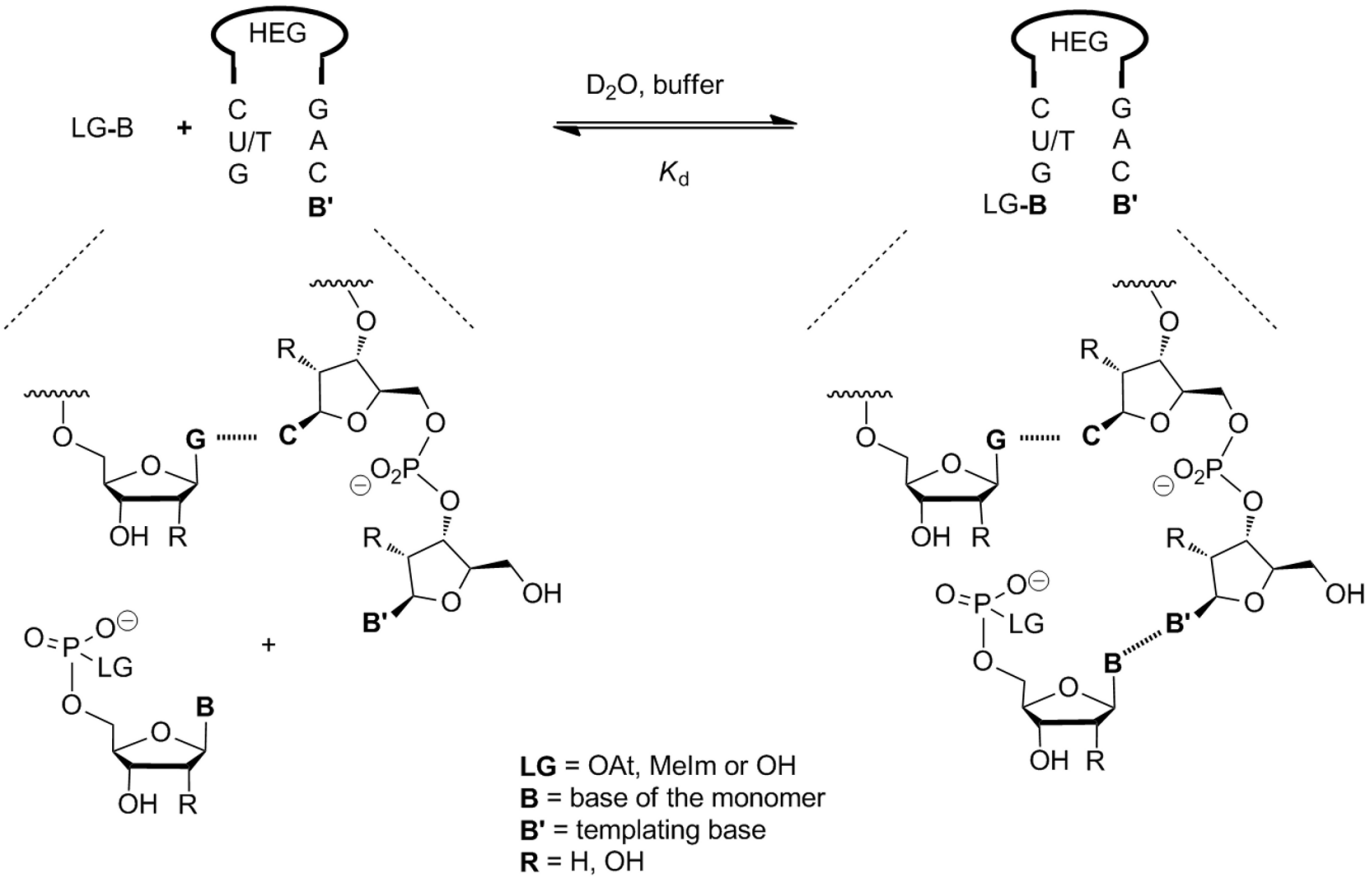
Binding equilibrium between mononucleotides and hairpins representing primer–template duplexes, as chosen for measuring dissociation constants by NMR titration.

While NMR spectroscopy is sensitive when performed with a modern high field-spectrometer, it does have the disadvantage of producing complex spectra that require detailed analysis to assign at least the most critical resonances unambiguously. This is why we chose small hairpins with a non-nucleosidic hexaethyleneglycol loop [44] for our NMR-monitored titrations (Figure 8). The Szostak group later measured binding constants for complexes of three of the four unactivated ribonucleotides (A, C, and G) by NMR using longer constructs [45]. The hairpins are stable at room temperature and consist of only seven nucleotides, facilitating the interpretation of spectra. Resonances of the nucleobases at the terminus with the templating base were readily identified. Dissociation constants were determined by fitting the chemical shifts of terminal nucleotides in the ^1^H NMR, measured at different nucleotide concentrations.

With the DNA hairpins and unactivated deoxynucleotides, depending on the sequence and experimental conditions, dissociation constants ranging from 10 mM (dGMP) and 280 mM (TMP) were measured [33–34]. In the RNA systems, the values measured were between 14 mM (GMP) and >500 mM (UMP). These results are similar to those obtained by Szostak and co-workers [45], who found that CMP binds most strongly, however, when studying a different sequence context.

We also measured *K*_d_ values for activated nucleotides, either with 2-methylimidazole or with oxyazabenzotriazole as leaving group using rapid NMR titrations to avoid hydrolysis. In the case of the DNA system, a largely unreactive natural deoxyribonucleotide at the 3'-terminus was used, not an aminodideoxynucleotide to prevent complications from reactions taking place during the titration. For RNA systems, there were also no significant signs of conversion on the time scale of our one-dimensional NMR experiments. The methylimidazole leaving group did not show a significant effect on the affinity of the nucleotides for the hairpins [34]. In contrast, the OAt group did lead to slightly stronger binding, and the effect was greatest for OAt-TMP and OAt-UMP, with a decrease in *K*_d_ by a factor of four to ten. Nevertheless, for strongly pairing bases, the difference between dissociation constants of free and activated monomers was minimal.

Besides NMR titrations with hairpins, we also used a different experimental approach to determine binding or dissociation constants. The complementary technique utilized the inhibitory effect of free nucleotides on primer extension. By adding increasing concentrations of the free nucleotide to the assays solutions and measuring the kinetics of extension, we were able to quantify binding independently, using global fits to the data sets. Here, longer templates were used, as well as downstream-binding oligonucleotides. Thus, 25 different dissociation constants were measured for different sequence contexts, ranging from 2 mM for dGMP and 200 mM for TMP [33]. This confirmed the positive effect of downstream-binding strands that we first reported in 2005 [28–29]. In the RNA case, we had shown that the presence of a 'helper strand' that is only three bases long can increase the yield of the extension by a factor of three at room temperature and by a factor of six in the cold. In their recent work, Szostak and co-workers measured dissociation constants for complexes of CMP via isothermal titration calorimetry [46]. When a downstream-binding strand was present, binding of the monomer was up to two orders of magnitude tighter than in its absence.

### Simulating primer extension

With dissociation constants in hand, we were now in a position to determine rate constants for the covalent step of primer extension. For each case, the concentration of the kinetically relevant species (the monomer–primer–template complex), i.e., the occupancy of the extension site by the monomer, was now known, and measuring the initial rates led to the *k*_cov_ value via fitting. For OAt esters and an aminoterminal primer on a DNA template, values of 2–10 h^−1^ were found, whereas methylimidazolides gave rate constants between 0.3 and 1.4 h^−1^ [34]. For TMP, the reactivity with an OAt leaving group is four-fold higher than with a MeIm leaving group. The largest increase in reactivity was found for dCMP whose reaction with the aminoprimer in the kinetically relevant complex is 25-fold faster as oxyazabenzotriazolide than as 2-methylimidazolide. For RNA primers on an RNA template, the values were between 0.01 h^−1^ (MeIm-AMP) and 0.1 h^−1^ (OAt-GMP). Overall, depending on the backbone, primer terminus, base, and leaving group, the rates of the chemical step vary by two orders of magnitude.

**Figure 9:**
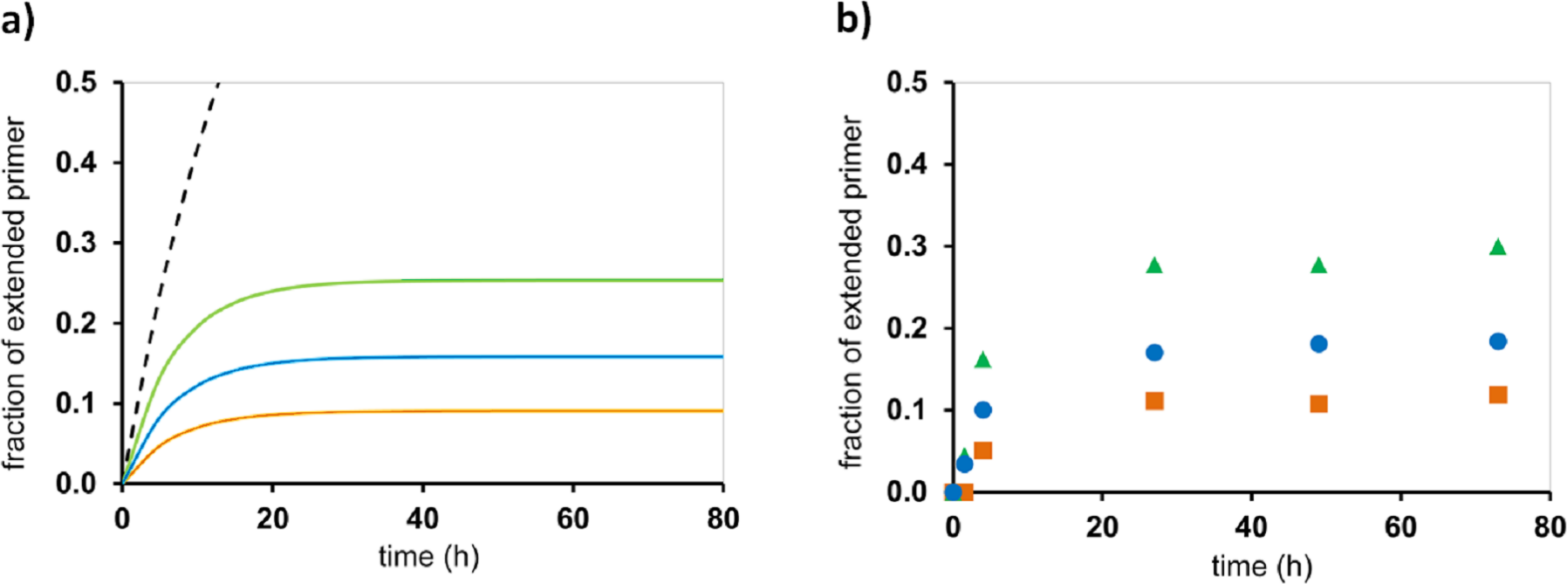
Template-directed primer extension on an RNA template performed with OAt-GMP at 1.8 mM (orange), 3.6 mM (blue), or 7.2 mM (green) initial concentration. a) Conversion over time, as simulated with our quantitative model, using the dissociation constants of both activated and free nucleotide, and rate constants for hydrolysis and chemical step. The broken black line is the hypothetical conversion of the primer without hydrolysis of monomer and the resulting inhibition; b) Corresponding experimental data, acquired in primer extension assays at 20 °C in buffer (200 mM HEPES, 400 mM NaCl, 80 mM MgCl_2_, at pH 8.9) at 36 µM primer–template (5'-UAUGCUGG-3' – 3'-CACCCACCACAUACGACCCAAGCACAC-5'); see reference [34] for further details.

Using the set of four constants, one may then calculate the time-dependent yield of primer extension using the mathematical form of the model shown in Figure 7 [33]. The data predicted by our model agreed quite well with experimental data for either of the four nucleobases and the two different backbone chemistries. Figure 9a and 9b show representative plots of theoretical yields and data points from RNA-based assays at different monomer concentrations. It can be discerned that 7.2 mM monomer concentration does not suffice to induce more than approx. 30% conversion of the primer. The theoretical data on the left also shows the calculated time–yield curve for a hypothetical assay that does not suffer from inhibition by spent monomers. For such a scenario, full conversion is expected to occur. This is in agreement with the experimental observation that periodic removal of spent monomers prevents the stalling of primer extension that otherwise plagues these assays [32].

**Figure 10:**
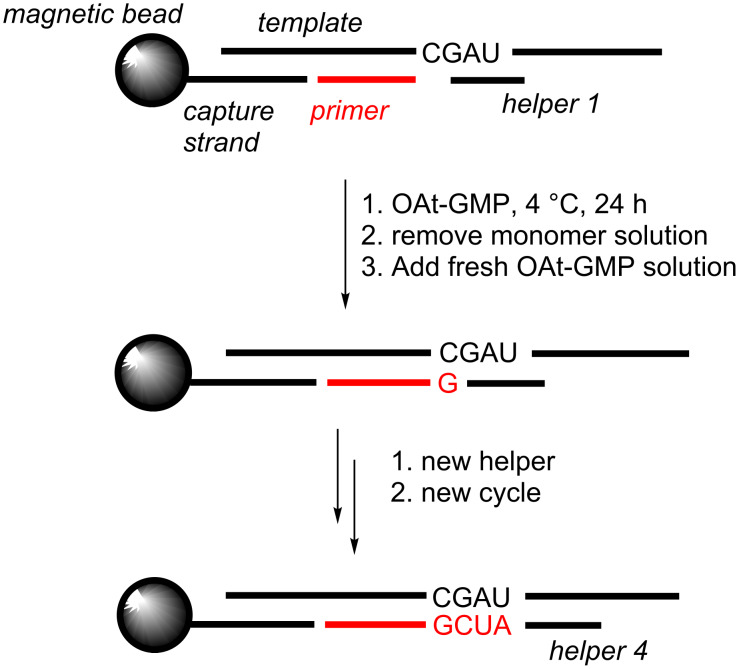
Copying of four nucleotides on an immobilized RNA duplex, as reported by Deck et al. [32].

As explained in more detail in reference [33], there are three extreme cases. In the first case, both primer and tightly binding monomer are so reactive that full conversion is achieved before inhibition can become significant. This is the scenario found for OAt esters and the aminoterminal primer. The second scenario involves reactive monomer and primer, but the monomer is binding poorly (e.g., TMP), with just a few percent occupation of the extension site at the beginning of the assay. Here, hydrolysis does catch up with the desired reaction eventually, but it is inconsequently, because the low occupancy does not produce a significant level of competitive inhibition. In other words, if there is not much of a template effect to begin with, the spent monomers will not outcompete the monomer over time, and the reaction will largely proceed as expected for a second-order reaction with a competing reaction that just drains active monomer (hydrolysis). In the third case the primer is fairly unreactive, being equipped with just the terminal diol of natural RNA. Further, the monomer is a strongly binding one (OAt-GMP). In this case, inhibition becomes significant over time, and removal or re-activation of the monomer is required to prevent the extension from ceasing before near-quantitative conversion is achieved. This is what was done in the successful copying assays with immobilized primer–template duplex (Figure 10).

The insights gained from the quantitative analysis of primer extension leaves several options to push assays to completion. The first is to employ highly reactive and well binding monomers only. For RNA, this approach does not appear realistic, if one wants to work with any given sequence context and all four bases (A/C/G/U). The second option is to remove the spent monomers when their concentration reaches a critical threshold. This requires immobilization of the primer–template complex and washing [32,47] or removal of hydrolyzed monomers by dialysis [38]. The third option is finding conditions for in situ activation, so that spent monomers can be re-activated during the time course of the assay. The fourth option is searching for better leaving groups that give a more favorable ratio of rates for primer extension and hydrolysis (*k*_cov_/*k*_hydr_), when reacting with an RNA primer. It will not be trivial to find such a leaving group, as the nucleophilicity of alcohols is quite similar to that of water, so that it is difficult to utilize the chemoselectivity toward reaction partners with different softness, p*K*_a_, or other structural features.

### Copying on solid support

As mentioned above, one option to avoid stalling of primer extension reactions is to perform them on solid support. For RNA, the immobilization of the primer–template duplex was achieved by employing a biotinylated capture strand that was bound to streptavidin-coated magnetic beads (Figure 10) [32]. The assays allowed for near-quantitative incorporation of any of the four nucleobases opposite their complementary base in the template, but the reactions on the RNA-based system are quite slow.

**Figure 11:**
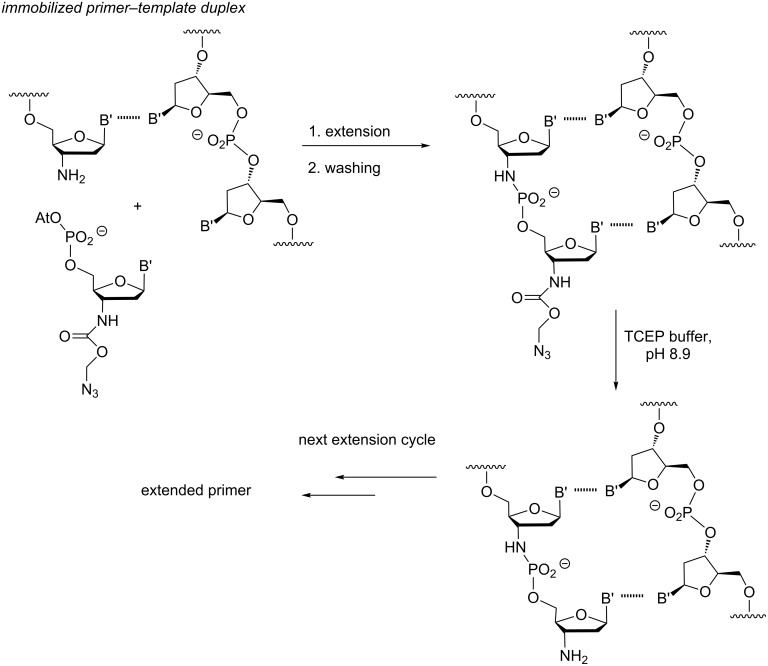
Extension cycle of aminoterminal primer with N-protected nucleotides on solid support, as described by Kaiser et al. [47].

For aminoterminal DNA, a methodology was developed by us that allows repeated incorporation of reactive 3'-amino-2',3'-dideoxynucleotide building blocks, activated as OAt esters [47]. This methodology can, in principle, be automated, and was established with a view towards sequencing, using fluorophore-labeled nucleotides [48–49]. To avoid cyclization or oligomerization of the monomer, the 3'-amine was protected with an azidomethyloxycarbonyl (Azoc) protecting group. This protecting group can be rapidly removed under non-denaturing conditions after incorporation by the complementary nucleotide using the Staudinger reaction with a water-soluble phosphine (Figure 11). This protocol, with what in the sequencing community is called "reversible termination", allowed efficient copying with any of the four nucleobases (A/C/G/T) in less than 12 h for each incorporation at room temperature. It was also used to demonstrate enzyme-free, template-directed primer extension in the non-natural direction (P3'→N5'), using 3'-phosphates of 3'-amino-2',3'-dideoxynucleosides [47].

### Activation chemistry

#### Imidazolium bisphosphates

**Figure 12:**
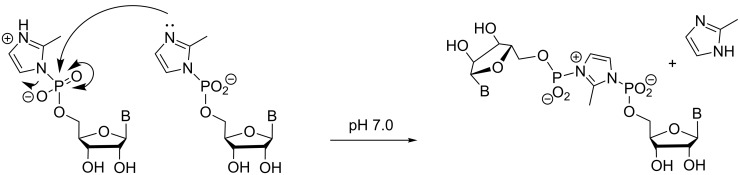
Formation of a highly reactive methylimidazolium bisphosphate from methylimidazolides of nucleotides.

**Figure 13:**
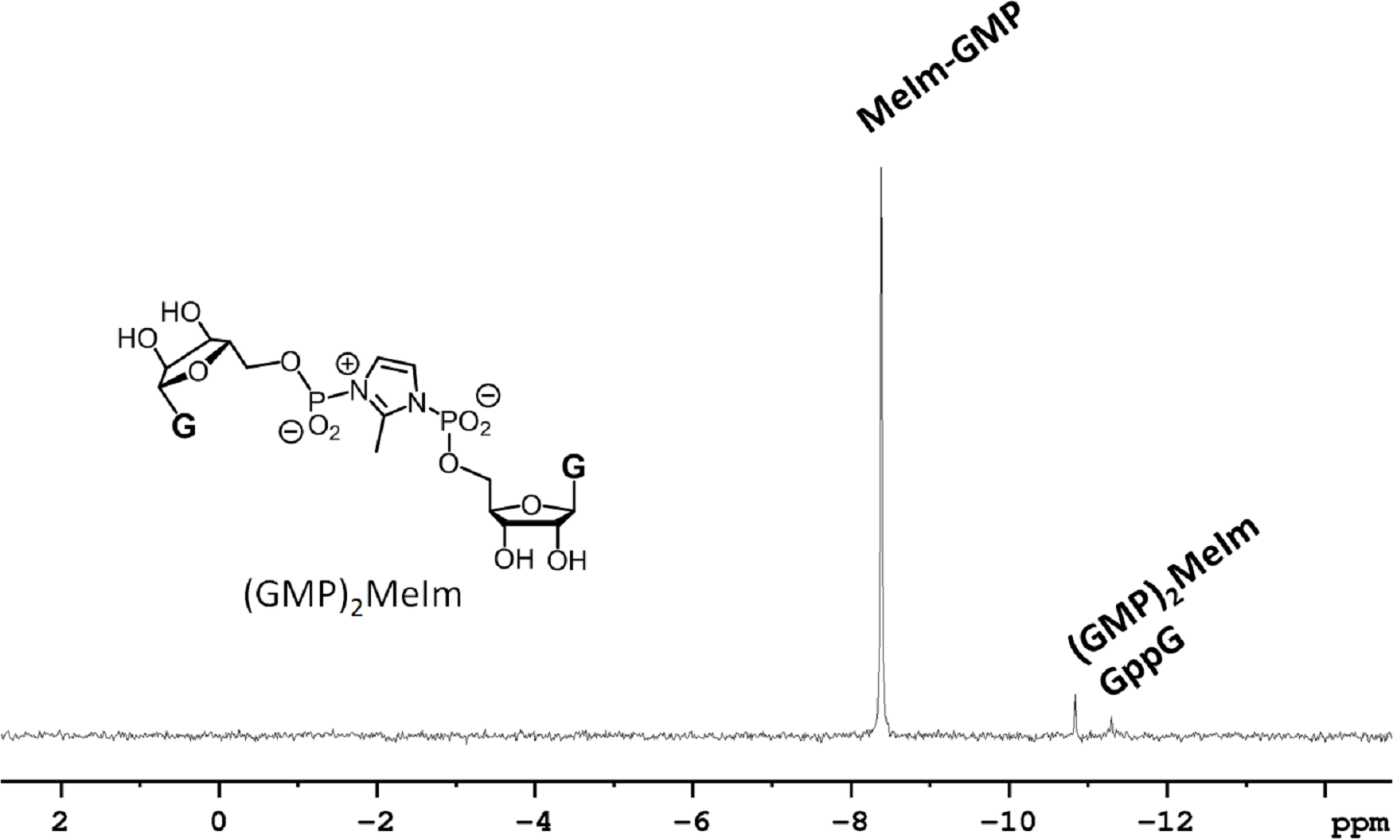
^31^P NMR spectrum (161.9 MHz) of crude MeIm-GMP in D_2_O. The resonance of the imidazolium bisphosphate appears at −10.8 ppm, and that of the pyrophosphate GppG at −11.3 ppm.

During our work on the effect of leaving groups on the yield of primer extension reactions, we noticed a burst phase in the kinetics of methylimidazolides that was only observed with monomers that were not carefully purified. The high reactivity was traced to a species with a chemical shift of −10.8 ppm in the ^31^P NMR spectrum that was identified as the imidazolium bisphosphate (Figure 12 and Figure 13) [34]. We calculated a second order rate constant for the reaction of the imidazolium bisphosphate with the primer of 2.9 × 10^4^ M^−1^ h^−1^, which is approx. 600-fold larger than that of the pure methylimidazolide. The kinetics and analytical data were presented in the Supporting Information of ref. [34]. Shortly afterwards, Szostak and co-workers published a series of papers on the role of imidazolium bisphosphates in primer extension [50–53], including NMR data for ^13^C-labeled 2-methylimidazolides that showed bonding to two phosphates. We did not pursue the imidazolium bisphosphate further because we did not observe full conversion of the primer at reasonable concentrations of this labile species. Other imidazolium phosphates, such as those formed upon in situ activation appeared more promising (vide infra).

**Figure 14:**
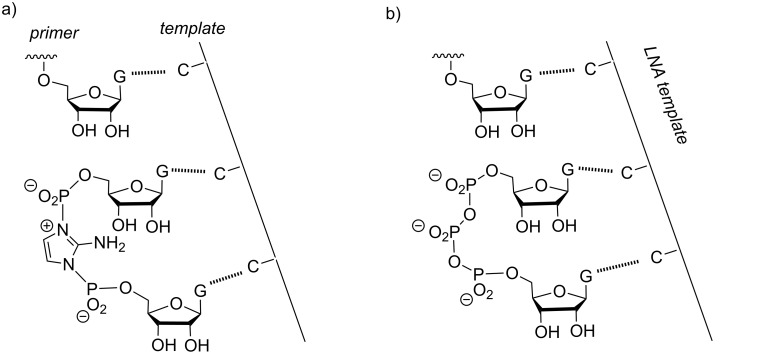
Imidazolium bisphosphate as intermediate in the primer extension reaction, as described by Szostak and colleagues. a) Intermediate of an extension with aminoimidazolides as monomers [52]; b) one of the structural arrangements found in a recent crystallography study that used oligophosphates as model compounds [53].

The extensive work on imidazolium bisphosphates by the Szostak group was prompted by an observation made during assays with a trimer downstream of the primer extension site, pre-activated as methylimidazolide. The presence of the leaving group was found to accelerate the incorporation reaction [51]. A subsequent optimization identified 2-aminoimidazolides as monomers with superior properties [50]. Figure 14a shows the proposed intermediate forming when two neighboring monomers have reacted, and Figure 14b shows the binding mode of a GpppG dimer that was found to bind in a fashion described as structurally similar to the proposed intermediate shown on the left-hand side [53]. In the latter case, LNA residues were used in the template strand to facilitate crystallization.

We note that the neighboring group participation proposed should be limited to leaving groups with a second nucleophilic site at the appropriate position. Other leaving groups than methylimidazole should not be able to react via the same dominant reaction pathway. In our hands, compounds with a different structure such as OAt esters give rapid and high-yielding reactions. This is also true for the intermediates of extension with in situ activation, which lack the second nucleophilic group entirely (vide infra). Further, we have consistently found that helper oligonucleotides without a phosphate group at the 5'-terminus accelerate primer extension reactions, both for aminoterminal primers [28] and for RNA-based systems [29]. If formation of an imidazolium bisphosphate was the dominant reaction pathway, this should not be the case. Without a phosphate group, the bisphosphate cannot be formed, and the helper should block the reaction pathway that requires this species bound to the template. Full conversion was found with OAt esters in the presence of an unphosphorylated helper, even for UMP [40]. The aminoimidazolium phosphates are interesting and well-binding species. Time will tell whether they provide the most favorable pathway for primer extension. Perhaps, the successful copying of long stretches of RNA templates will be the ultimate test for their ability to support enzyme-free copying.

#### In situ activation

Re-activation of hydrolyzed monomers during the course of the extension assay is another approach to avoid stalling due to inhibition. As mentioned in the Introduction, ligation reactions had been achieved by Naylor and Gilham in aqueous media in presence of a water-soluble carbodiimide as condensing reagent [7]. Likewise, Sulston et al. had used EDC to oligomerize AMP in the presence of poly(U) as template [12]. Polymerization of nucleotides with in situ activation had also been attempted with the aid of montmorillonite, a clay mineral, but had led mostly to dimers and pyrophosphate [54]. For DNA, ligations starting from unactivated starting materials were known [10,55–56], but not always high-yielding, unless an aminoterminal strand was reacted with the phosphate-terminated counterpart [57–59], to form a phosphoramidate-linked product. Efficient versions of extension of an RNA primer with in situ activation were not known to us.

One difficulty in inducing the extension of RNA primers with ribonucleotides without preactivation lies in the different pH optima of the two reactions. The activation, which now has to occur in the same solution as the extension, is most easily performed under slightly acidic conditions, whereas the extension reaction is favored under basic conditions, particularly when good leaving groups, such as oxyazabenzotriazolides are involved. Further, the activating agent (condensing agent) is an electrophile, and there is significant potential for side reactions of the reagent with other nucleophilic groups than the 5'-phosphate of the desired nucleotide. As a consequence, assays involving in situ activation were slow and low yielding.

We assumed that the inefficient primer extension with in situ activation could be improved via organocatalysis. We had previously found, when studying extension of aminoterminal primers, that small heterocycles, such as pyridine, can increase the rate of the reaction [60]. Most probably, this effect was organocatalytic in nature, being caused by a pyridinium species that forms in the reaction medium, an effect known from the acceleration of DCC-induced acylation reactions with dimethylaminopyridine [61]. With aminoterminal primers, in situ activation and organocatalysis with 1-methylimidazole in a magnesium-free buffer had led to encouraging results, even at submillimolar nucleotide concentration [62]. So, starting from a primer extension reaction with an RNA-system that gave less than 1% conversion after 24 h, a number of heterocycles were screened. The best results were obtained for 1-methyladenine and 1-ethylimidazole (1-EtIm) [63]. Optimization of the reaction conditions then led to a method that gave 90% conversion in 48 h, successful incorporation of more than one nucleotide in a row, and high yielding extension even with poorly binding UMP, all under the same conditions. We dubbed these conditions "general condensation buffer". The optimized buffer contains 500 mM HEPES, 800 mM EDC, 80 mM MgCl_2_, and 150 mM 1-EtIm. Assays are usually performed at 0 °C to shift the binding equilibrium for the incoming nucleotide to the bound side, and thus strengthen the template effect.

**Figure 15:**
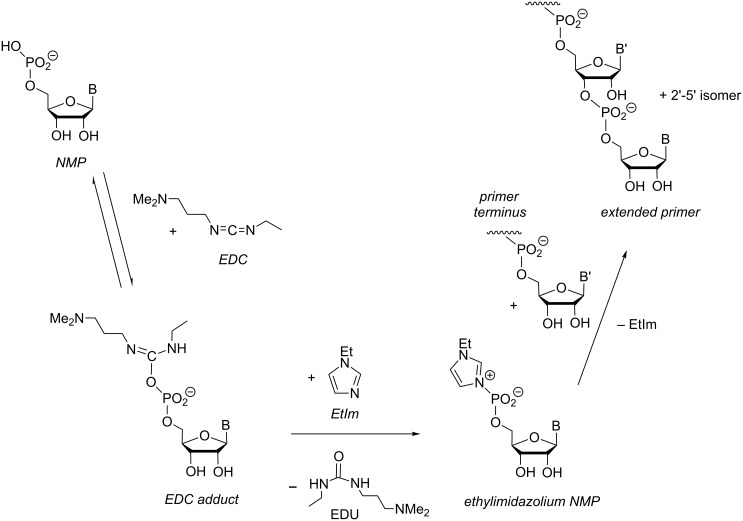
Proposed steps of enzyme-free primer extension with in situ activation, using the "general condensation buffer" containing EDC and 1-ethylimidazole as organocatalyst.

The proposed mechanism for the reaction is shown in Figure 15. In order to start the activation, the carbodiimide has to react with the phosphate group, leading to what is sometimes called a "covalent adduct". This first step may either occur in solution, or while the nucleotide is already bound to the primer–template duplex. We have not been able to observe a signal for the "EDC adduct" in the NMR spectra, and the binding equilibrium establishes itself quickly. We assume that the on- and off-rate are much faster than the NMR time scale. The EDC adduct is then expected to react with the organocatalyst, yielding the alkylimidazolium nucleotide that acts as the kinetically most relevant monomer in the extension reaction. The ethylimidazolium species can be observed as a small peak in ^31^P NMR spectra. The extension occurs as expected, most probably via addition/elimination, including a pentavalent intermediate and possibly by a pseudorotation to place the ethylimidazole leaving group in an apical position. Since either of the two alcohols of the terminal diol of the primer can attack, a mixture of 3',5'- and 2',5'-isomers is expected for this reaction. Work on quantifying the ratio of the diastereomers formed is under way in our laboratories.

Unlike assays performed with pre-activated monomers, primer extension with in situ activation of ribonucleotides led to significant levels of oligomers that form via untemplated polymerization of the nucleotides [63]. This was a welcome side reaction, as it helps to explain how RNA may have been formed and copied under prebiotic conditions. In fact, when oligomerization assays were performed with any of the four natural ribonucleotides, oligomers of mixed sequence long enough to hybridize stably to complementary strands were formed. Such strands may then be the templates or primers required to start enzyme-free copying.

Further, the general condensation buffer noted above gave rise to the spontaneous formation of ribonucleotide- or RNA-linked peptides [64]. These peptides are linked via their N-terminus to the ribonucleotide portion as phosphoramidates, which is why we refer to them as "peptido RNAs". Peptide chain growth on the 5'-phosphate is much faster than the background reaction [65], and will thus predominate over background oligomerization of amino acids alone. Further, the rate of formation of peptido RNA depends on the structure of the amino acid, and, to a lesser degree, on that of the ribonucleotide [66], so that a very primitive, not yet encoded form of RNA-induced peptide synthesis can occur under conditions that support the formation and copying of genetic information. We felt that this was significant for theories on the emergence of life from inanimate materials, even more so as the same reaction conditions also support the formation of pivotal cofactors of primary metabolism from nucleotide precursors [64]. The reactions mentioned above occur spontaneously in cold aqueous solution, without the need for mineral surfaces or enzymes.

Condensation producing peptido nucleotides also occurs with other activating agents, such as cyanamide or carbonyl diimidazole (CDI). Cyanamide, a tautomer of unsubstituted carbodiimide, has long been considered a prebiotically relevant activating agent [67], and it has previously been used in experiments aimed at generating peptides in the absence of enzymes or a ribosomal machinery [68]. Reactions with cyanamide are much less efficient than with EDC, so that successful primer extension has not yet been observed in our assays. But, while reactions that take weeks or months are not problematic for prebiotic evolution, which probably occurred over many millions of years, they are difficult to study in detail in an academic setting that requires results on the time scale of Ph.D. theses.

## Conclusion

Enzyme-free primer extension is a fascinating reaction that has been linked to the origin of the first self-replicating systems. The reaction does produce extended primers with nucleotides complementary to the template sequence being appended at the 3'-terminus, but it is slow and low-yielding, particularly when performed with natural RNA/ribonucleotides. Because it relies on weak Watson–Crick base pairing between a single nucleotide and a templating base, the reaction cannot be driven to completion by heating or harsh conditions. Instead, a subtle interplay of binding equilibria and chemical steps either leads to successful incorporation of the nucleotide monomer or to the more likely path of hydrolysis, which in turn can prevent further extension via competitive inhibition [69]. Detailed quantitative work has led to a better understanding of the processes underlying incomplete conversion and thus to approaches that reduce inhibition or slow conversion. Among them is the removal of hydrolyzed monomer, improved activation chemistries, or in situ (re)activation with the support of an organocatalyst. Despite progress in the field, the ultimate goal of demonstrating enzyme-free replication of RNA strands long enough to code for an oligo- or polypeptide is not yet in sight. This is particularly true, if one considers that the issue of low sequence fidelity was not even discussed in this short account. Much remains to be done for chemists and biochemists alike.
